# Diffuse Alveolar Hemorrhage: An Uncommon Manifestation of Vaping-associated Lung Injury

**DOI:** 10.7759/cureus.6519

**Published:** 2019-12-31

**Authors:** Rodger Wilhite, Tarang Patel, Ethan Karle, Shyam Shankar, Armin Krvavac

**Affiliations:** 1 Pulmonary, Critical Care and Environmental Medicine, University of Missouri Health Care, Columbia, USA; 2 Internal Medicine, University of Missouri Health Care, Columbia, USA; 3 Pulmonary and Critical Care Medicine, University of Missouri Health Care, Columbia, USA

**Keywords:** inhalational lung injury, diffuse alveolar hemorrhage, pulmonary medicine, electronic cigarette, tetrahydrocannabinol, pulmonary embolism, evali, pneumonitis, vaping-induced lung injury, vaping-associated lung injury

## Abstract

Vaping involves the use of a device to deliver aerosolized nicotine and tetrahydrocannabinol (THC) oils to the lungs. Vaping continues to increase in popularity; however, because it is a novel drug delivery system there is little evidence regarding its safety and long-term consequences. Here, we present a 22-year-old Caucasian male who was admitted with acute hypoxic respiratory failure and massive hemoptysis. Contrasted computed tomography of the chest demonstrated ground glass opacities throughout all lung fields and bilateral pulmonary emboli. Bronchoalveolar lavage revealed increased red blood cell counts in serial aliquots, consistent with the diagnosis of diffuse alveolar hemorrhage (DAH). An extensive workup did not reveal an etiology for the DAH. However, further history was obtained, and the patient divulged daily vaping of THC. E-cigarette, or vaping, product use associated lung injury (EVALI) consists of a myriad of different lung injury patterns. Our case illustrates an uncommon presentation of EVALI with DAH and multiple pulmonary emboli.

## Introduction

Electronic cigarette (e-cigarette) use or vaping involves the use of a device to heat liquids in order to deliver an aerosolized product to the lungs. E-cigarette, or vaping, product use associated lung injury (EVALI) has reached epidemic proportions with 1,888 reported cases and 37 deaths related to vaping [1]. EVALI has become a public health crisis threatening our nation’s youth due to marketing directed towards adolescents and easy modification of devices that allows the introduction of tetrahydrocannabinol (THC) oils [2,3]. The diagnosis of EVALI is difficult to elucidate as a myriad of radiographic patterns including acute eosinophilic pneumonia, organizing pneumonia, lipoid pneumonia, diffuse alveolar damage, hypersensitivity pneumonitis, and diffuse alveolar hemorrhage (DAH) have been described [4]. We illustrate a patient with EVALI that presents with DAH and pulmonary embolism.

## Case presentation

A 22-year-old Caucasian male with no significant medical history was admitted for acute hypoxemic respiratory failure and massive hemoptysis. Physical examination revealed an afebrile and tachycardic young male in severe respiratory distress, oxygen saturating of 89% on 15 liters per minute of supplemental oxygen via a non-rebreather mask. He exhibited use of accessory respiratory muscles and diffuse rhonchi bilaterally. He underwent emergent endotracheal intubation in the setting of massive hemoptysis. Laboratory testing revealed neutrophil predominant (83.9%) leukocytosis of 27.82 x10^9^/L, hemoglobin of 12.7 g/dL, and thrombocytopenia of 129 x10^9^/L. Electrolytes, renal function, liver enzymes, and coagulation studies were all within normal limits. Procalcitonin, N-terminal pro B-type natriuretic peptide, and C-reactive protein were elevated at 0.92 ng/mL, 634 pg/mL, and 16.04 mg/dL respectively. Lactic acid was normal at 1.5 mmol/L. The urine drug screen was positive for cannabinoids.

**Figure 1:**
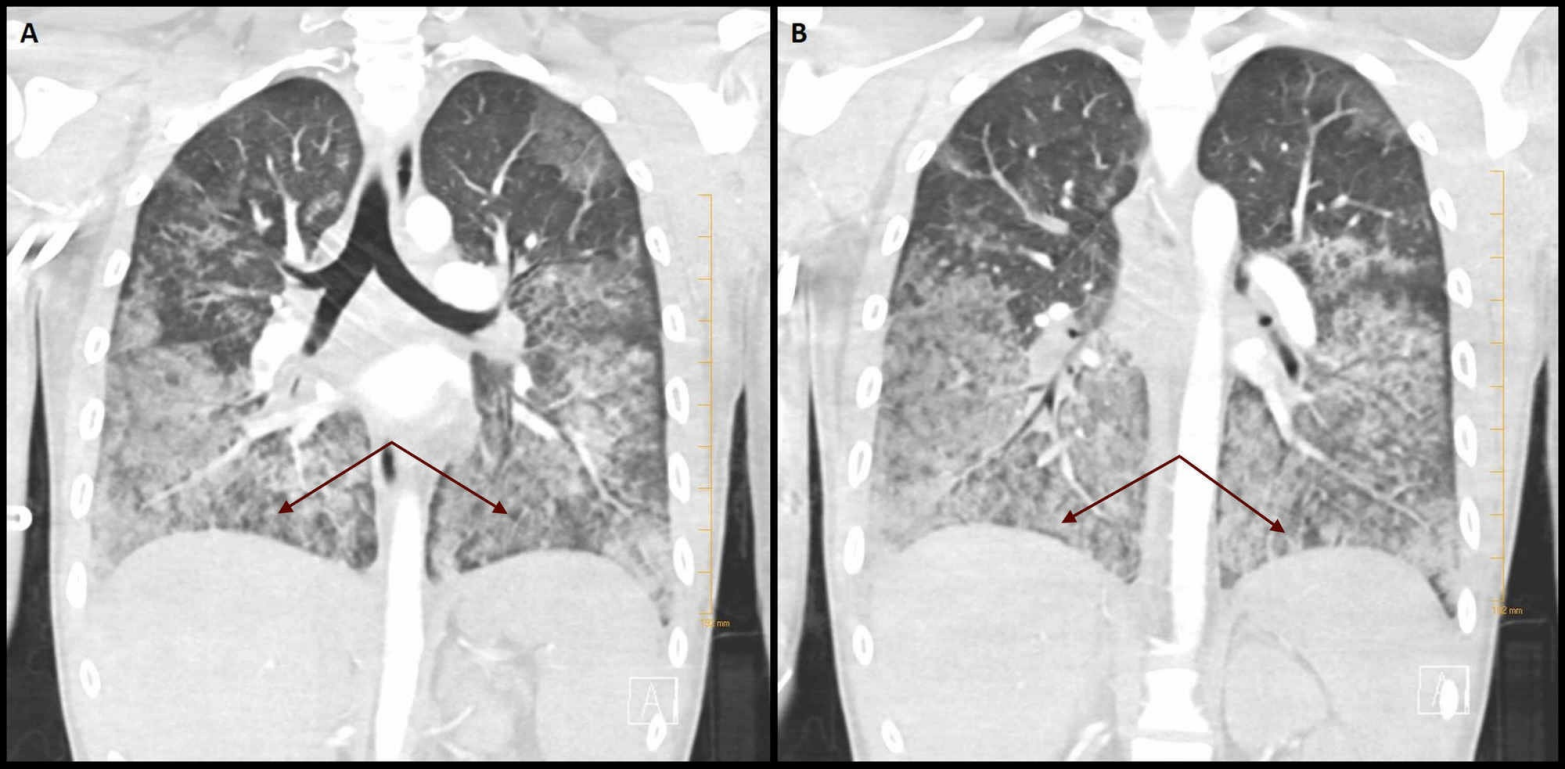
Computed tomography of the chest. (A, B) Coronal section of computed tomography of the chest (arrows) demonstrating bilateral lower lobe predominant ground-glass opacities.

**Figure 2:**
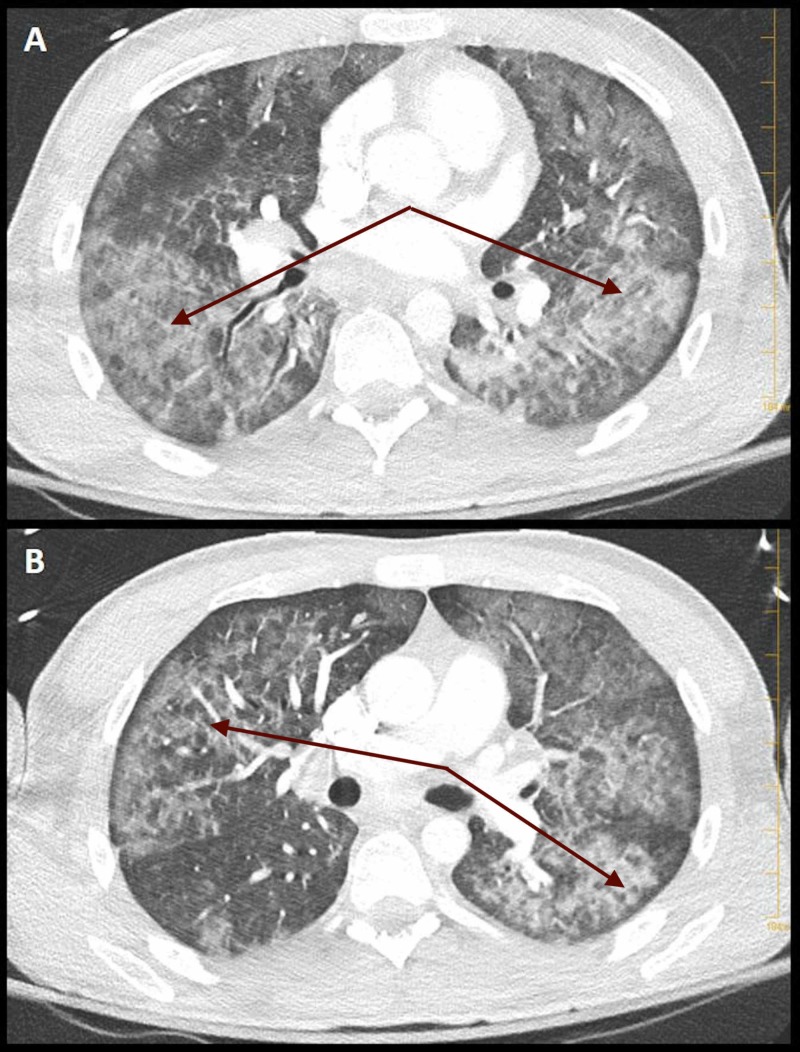
Computed tomography of the chest. (A, B) Axial section of computed tomography of the chest (arrows) demonstrating bilateral ground-glass opacities.

A computed tomography (CT) scan of the chest was obtained and demonstrated diffuse ground-glass opacities and multiple pulmonary emboli (Figures 1, 2).

The patient underwent bronchoscopy and bronchoalveolar lavage, which revealed increasing red blood cells in three serial aliquots, consistent with DAH. Further investigation to explicate the etiology for DAH was unremarkable, and workup for autoimmune diseases, infectious causes, hematologic, and oncologic causes was negative (Tables 1-4).

**Table 1:** Autoimmune Diagnostic Evaluation of Diffuse Alveolar Hemorrhage Anti-ds-DNA, double stranded-deoxyribonucleic acid; ANA, anti-nuclear antibody; anti-PR3, anti-proteinase 3 antibody; anti-MPO, anti-myeloperoxidase antibody; anti-GBM, anti-glomerular basement membrane antibody; ACE, angiotensin-converting enzyme.

Test	Result
Anti-Ro	<4.9 RLU
Anti-La	<3.3 RLU
Scl-70	<1.2 RLU
Anti-Jo-1	<2.2 RLU
Anti-ds-DNA	Negative
ANA	Negative
Anti-PR3	<2.3 RLU
Anti-MPO	<3.2 RLU
Anti-GBM IgG	<2.9 RLU
ACE level	12 U/L

**Table 2:** Infectious Diagnostic Evaluation of Diffuse Alveolar Hemorrhage RPP, respiratory pathogens panel; HIV, human immunodeficiency virus; CMV, cytomegalovirus; RMSF, Rocky Mountain spotted fever; PCR, polymerase chain reaction; BAL, bronchoalveolar lavage; AFB, acid-fast bacilli.

Test	Result
RPP	Non-reactive
Sputum cultures	Negative fungal, bacterial, AFB
Blood cultures	Negative fungal, bacterial, AFB
Urine streptococcal antigen	Negative
Urine legionella antigen	Negative
Urine histoplasma antigen	Negative
HIV	Non-reactive
Hantavirus antibody	Negative
Anaplasma IgG	<1:64
CMV DNA	Not detected
Lyme disease titers IgG, IgM	Negative
RMSF titers IgG, IgM	<1:64
Ehrlichia PCR	Not detected
Fungal BAL antigen	Negative

**Table 3:** Neoplastic Diagnostic Evaluation of Diffuse Alveolar Hemorrhage FNA, fine-needle aspiration; BAL, bronchoalveolar lavage.

Test	Result
Peripheral blood smear	Negative for malignant cells
Lymph node FNA	Negative for malignant cells
Flow cytometry	Negative for malignant cells
Cytology BAL	Negative for malignant cells

**Table 4:** Hematologic Diagnostic Evaluation of Diffuse Alveolar Hemorrhage PT, prothrombin.

Test	Result
Factor V Leiden mutation analysis	Mutation not detected
PT 20210G > A mutation	Mutation not detected
Anti-cardiolipin antibody	<1 RLU
Protein C	81%
Protein S	91%
Lupus anticoagulant antibody	Not detected

The patient subsequently divulged routine vaping with THC containing products that were purchased off the streets. His clinical condition improved after initiation of methylprednisolone with eventually transition to an oral prednisone taper.

## Discussion

The recent recognition of EVALI is perplexing since vaping products were introduced in 2009. A major driver of the recent epidemic may be the increasing popularity of vaping among adolescents. Monitoring the future surveyed more than 40,000 adolescents and uncovered that the prevalence of vaping among them increased by more than twofold over the past two years [5]. Another contributor to the recent rise in cases may be the introduction of THC oils into vaping products. In the largest published case series of EVALI, 80% of the 53 patients reported vaping of THC oils [3]. Lastly, the variation in clinical symptoms and radiographic findings among various cases may have contributed to a lack of recognition prior to the recent epidemic, as case reports of e-cigarette-induced lung injury can be traced all the way to 2012 in the literature [6].

EVALI is reported most commonly among younger male patients, with presenting symptoms of fever, shortness of breath, and cough. Nearly all cases present with bilateral opacities on chest imaging, a neutrophil predominant leukocytosis >11,000/mm^3^, and an elevated erythrocyte sedimentation rate. Clinicians should consider EVALI as the cause of respiratory failure in adolescent patients who endorse a history of vaping and present with bilateral radiographic opacities on chest imaging [3]. Although most cases present with bilateral opacities, the reported patterns on CT scans of the chest are much more heterogeneous [7].

The variety of radiographic presentations suggests that the pathophysiology of EVALI may be multifactorial. EVALI has been associated with the accumulation of lipid-laden macrophages (LLMs), although it is unclear if LLMs represent a pathophysiologic cause of lung injury [8,9]. Additionally, vaping appears to be directly toxic via thermal injury as e-cigarette aerosols can reach temperatures extreme enough to damage the respiratory epithelium [10]. Analyses of urine samples of e-cigarette users have demonstrated elevated levels of heavy metals [11]. Lastly, propylene glycol and glycerol produced by vaping have been associated with increased distal airway injury [12]. Limited case reports have implicated heavy metals, propylene glycol, and glycerol in the propagation of DAH [13-15]. Furthermore, our patient presents with a pulmonary embolism. Although it is unclear if there is a relationship between vaping and the development of pulmonary embolism, we know that there is a clear association between tobacco use and hypercoagulability [16].

## Conclusions

At this time, the causative agent or agents and pathologic mechanisms of EVALI remain poorly understood. Our case exhibits an uncommon presentation of EVALI with DAH and multiple pulmonary emboli. It is unclear if there is a relationship between vaping and the development of pulmonary embolism. However, there is a clear association between tobacco use and hypercoagulability. Clinicians should continue to remain vigilant for new cases of EVALI given the various possible clinical, radiographic, and pathologic manifestations.
